# Supplementary material for: Association between oral microbiome and seven types of cancers in East Asian population: a two-sample Mendelian randomization analysis
Source: Front Mol Biosci. 2023 Nov 21;10:1327893. doi: 10.3389/fmolb.2023.1327893 (PMC10702768; doi:10.3389/fmolb.2023.1327893)
Supplement: Supplementary file 1 [file DataSheet1.ZIP › Supplementary Materials/MR plots for tongue/tongue═╝/Ovarian cancer/pheno.1308_to_ovarian cancer_leave_one_out.pdf]

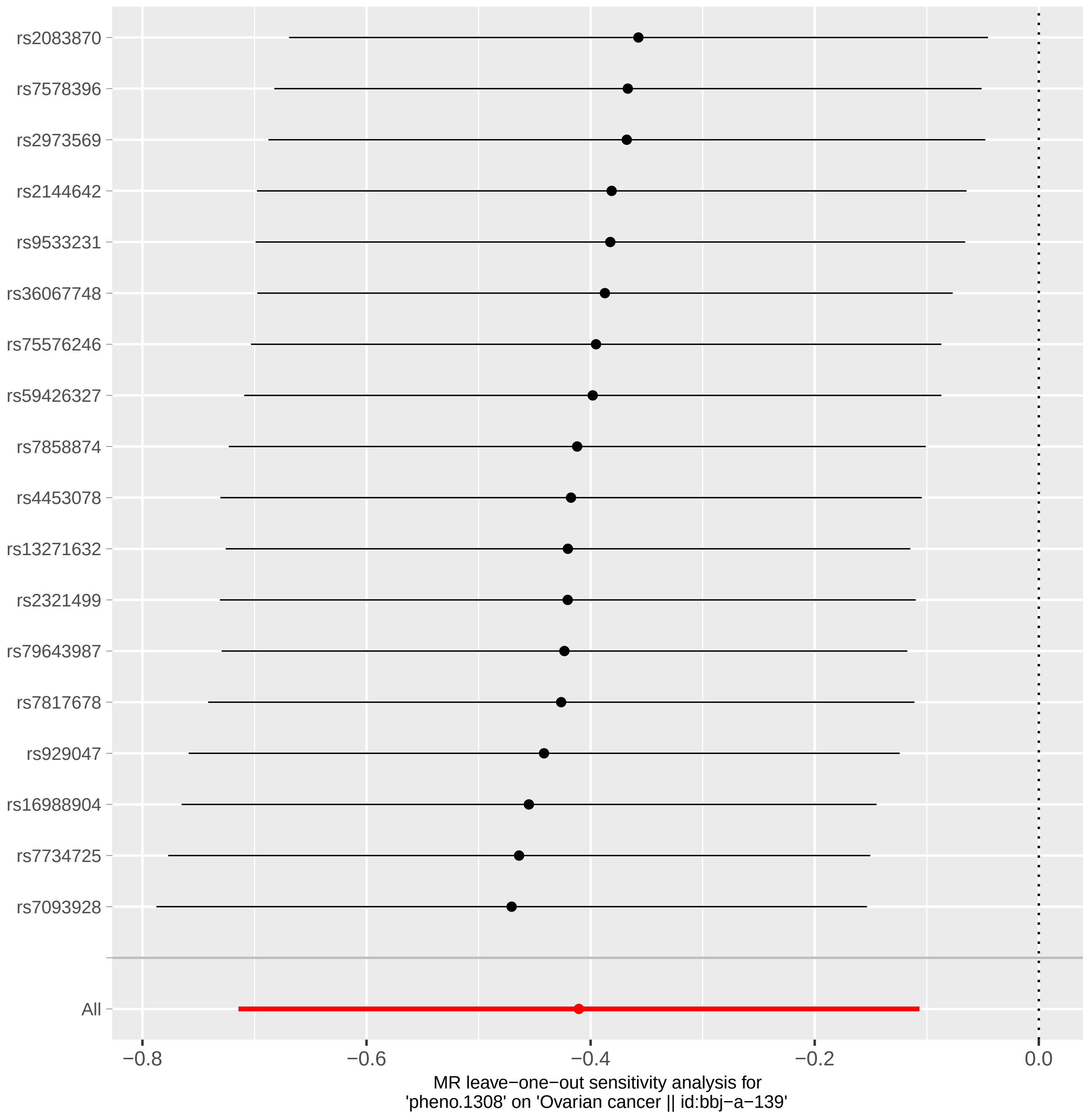
rs2083870
rs7578396
rs2973569
rs2144642
rs9533231
rs36067748
rs75576246
rs59426327
rs7858874
rs4453078
rs13271632
rs2321499
rs79643987
rs7817678
rs929047
rs16988904
rs7734725
rs7093928
All
−0.8
−0.6
−0.4
−0.2
0.0
MR leave−one−out sensitivity analysis for
'pheno.1308' on 'Ovarian cancer || id:bbj−a−139'
